# Validity and Reliability of Using a Belt-Worn Accelerometer on the Lower Back to Monitor Physical Activity

**DOI:** 10.3390/s26020429

**Published:** 2026-01-09

**Authors:** Sarah L. Williamson, Jin Luo, Ian P. Albery

**Affiliations:** 1 School of Science, Psychology, Arts and Humanities, Computing, Engineering and Sport, Canterbury Christ Church University, Canterbury CT1 1QU, UK; 2 School of Medicine and Biosciences, University of West London, London W5 5RF, UK; jin.luo@uwl.ac.uk; 3 School of Allied Health and Life Sciences, London South Bank University, Southwark, London SE1 0AA, UK; alberyip@lsbu.ac.uk

**Keywords:** accelerometer, physical activity, measurement, validation study, methodology

## Abstract

**Highlights:**

**What are the main findings?**

- The belt method is valid and reliable for collecting daily physical activity when tested against the laboratory standard.
- Participants can position sensors correctly themselves with the belt method by following a set of instructions.

**What is the implication of the main finding?**

- The belt-mounting method can be used in future low back pain studies for identifying a dose–response effect on low back pain outcomes, in terms of physical activity.
- This method is participant friendly, demonstrates good day-to-day reliability, and can be used in trials to collect physical activity data over an extended period of time.

**Abstract:**

This study evaluated the validity and reliability of physical activity measurements collected using a belt-mounted accelerometer worn at the lumbar spine. The study consisted of two parts, with 10 healthy participants in each part. In part 1, physical activity measurements collected from a belt-mounted accelerometer were compared with that from a skin-mounted accelerometer during controlled exercises and free-living activities, with both accelerometers worn simultaneously at the same anatomical location. In part 2 physical activity measurements in controlled exercises were compared between two different days, with either the belt-mounted accelerometer or skin-mounted accelerometer worn singularly. The results demonstrated no significant difference in physical activity measurements between either mounting method, or between the two testing days during controlled activities. These results indicate that the belt-mounting method is valid and has good day-to-day reliability and can be used in studies requiring long-term data collection to assess the impact of physical activity-related rehabilitation and low back pain.

## 1. Introduction

Physical activity (PA) is a staple recommendation by National and International Guidelines [1,2,3] as a vital ingredient of a healthy lifestyle and in the risk mitigation of chronic disease and disability [4,5], including low back pain (LBP) [1]. However, measuring PA levels in the general population is a challenging task, often relying on self-report measures [5]. Self-report measures have several limitations, such as the inability to provide absolute amounts of PA, overestimation of PA levels [6], being prone to recall bias [7], and the inability of the participants to accurately recall activity intensity [8]. Accelerometers offer a reliable, objective alternative to self-reported measures [9], making them widely used in research and medical settings for both adult and paediatric studies.

LBP is a musculoskeletal disorder that is often linked to excessive or improper loading of the spine and surrounding structures [10,11]. However, there is a sparsity in research using objective measures to quantify activity levels in an LBP population [12]. Accelerometers could be very useful in LBP research, as they could provide an estimation of loading on the spine from various activities in real-world conditions. In previous research, accelerometer data has been used to derive a range of parameters to estimate musculoskeletal load, such as daily step count, activity counts, activity intensity level, body posture [12], loading intensity, and loading dose [13]. These parameters could be useful for LBP researchers aiming to establish the effect or impact that certain quantities of activity have on outcomes of LBP, such as pain level or perceived disability [14].

Bone-mounted accelerometers are considered the “gold standard” in accurately measuring acceleration of the spine, but there are ethical issues with this approach [15]. In research settings accelerometers have generally been attached to the skin of lower back using double-sided, hypoallergenic sticky tape [16,17]. This method can measure acceleration with good accuracy if the soft tissue artefacts are minimised by careful mounting [18]. However, there have been several disadvantages reported with attaching the accelerometer to the skin, such as skin reactions and irritation, and requiring researchers to apply the accelerometer [19,20]. Compliance with wearing the skin-mounted accelerometer on the lower back is reported to be low, as seen in a study by Schneller et al. [19] where only 59.5% of participants adhered to the protocol (24 h/day for seven consecutive days). As PA is recommended as a self-management strategy for people with LBP [1], there is a need for a reliable and accurate method to measure PA over a prolonged period of time. To facilitate long-term monitoring of PA, an alternative method to the skin-mounted accelerometer is to attach the accelerometer to a removeable device, such as an elasticated belt, which can then be worn on the lower back [21], i.e., a belt-mounting method. The lower back is suggested to be a stable mounting area, experiencing less movement and being less subjected to interference [22]. Belt-mounted accelerometers have often been used in trials monitoring PA levels in children [5,23,24]. A scoping review of objective measures of PA in LBP research indicates that only a small portion of the included trials (8 of the 39 studies, (20.25%)) used belt-mounted accelerometers on the lower back to assess the PA levels [12]. These studies did not involve long-term monitoring of PA in free-living environment, with a few trials only collecting PA data over the course of 7 days [25,26,27,28] or up to 2 weeks [29,30]. During these trials, the participants were asked to mount accelerometers by themselves. However, it is unclear from the trial protocols if the participants were shown, or provided with a set of instructions, on how to properly wear the accelerometer around their lower back. This is different from laboratory test conditions where the mounting can be strictly controlled by researchers. It is thus important to examine how reliable and accurate the belt-mounting method is in collecting PA data, especially when the participants apply the accelerometer themselves, compared with standard laboratory methods such as the skin-mounting method.

To the authors’ knowledge, there is currently a lack of published studies on the validity and reliability of wearing accelerometers around the waist, using an elasticated belt, for measuring PA levels. To fill this gap, we aimed to compare the two different methods (skin-mounting method vs. belt-mounting method) of wearing accelerometers on the lower back, during controlled activities and free-living (daily) activities. We also aimed to investigate whether self-mounting the belt by the participants themselves affects the reliability of the PA measurements.

## 2. Materials and Methods

### 2.1. Design

This study is divided into two parts. In part 1, the validity of the belt-mounting method was examined by comparing PA measurements between a skin-mounted accelerometer and a belt-mounted accelerometer when worn simultaneously at the same location (lumbar spine, spinal level L4–L5). In part 2, the reliability of the belt-mounting method was examined by comparing PA measurements from two occasions. On each occasion, participants were asked to apply and wear the belt independently, based on a set of instructions provided (see Appendix A).

The study was approved by the London South Bank University (LSBU) Ethics Committee (School of Applied Sciences: Part 1—Ref: SAS 1711, Part 2—Ref: SAS1810). Informed consent was obtained from all participants.

### 2.2. Participants

#### 2.2.1. Inclusion Criteria:

To be eligible for this study, participants had to be the following:

- Over the age of 18.
- Free of musculoskeletal injury or disability.
- Physically able to complete the set physical activities.

#### 2.2.2. Accelerometers

Three-axis accelerometers (23 × 32.5 × 7.6 (mm); AX3 logging sensor, Axivity Ltd., Newcastle, UK) were used to measure PA. The accelerometers were configured each time prior to data collection with a sampling frequency of 50 Hz, and a magnitude range of ±16 g, using AX3 GUI [V1.0.0.30] (The Open Movement Software, Newcastle University, UK). The height and weight of each participant were also input during the configuration of each accelerometer.

### 2.3. Accelerometer Mounting Methods:

#### 2.3.1. Skin-Mounting Method

**Figure 1:**
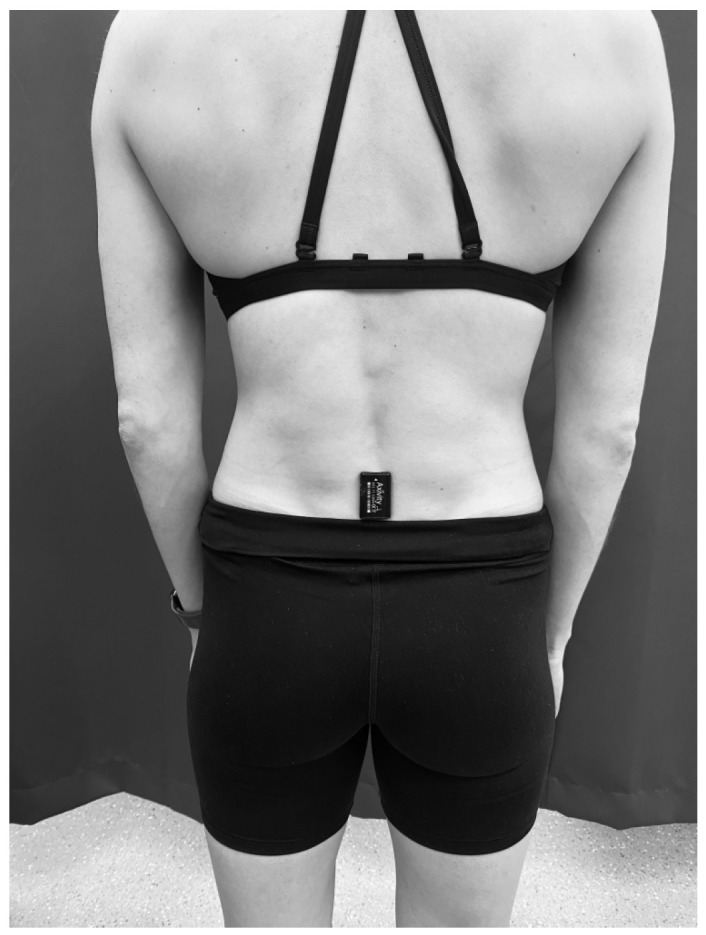
Skin-mounting method of attaching the accelerometer to the spinal level L4–L5 using double-sided hypoallergenic sticky tape.

The skin-mounting method consisted of using double-sided hypoallergenic sticky tape to attach the accelerometer to the participants’ lumbar spine in the L4–L5 region (see Figure 1).

#### 2.3.2. Belt-Mounting Method

**Figure 2:**
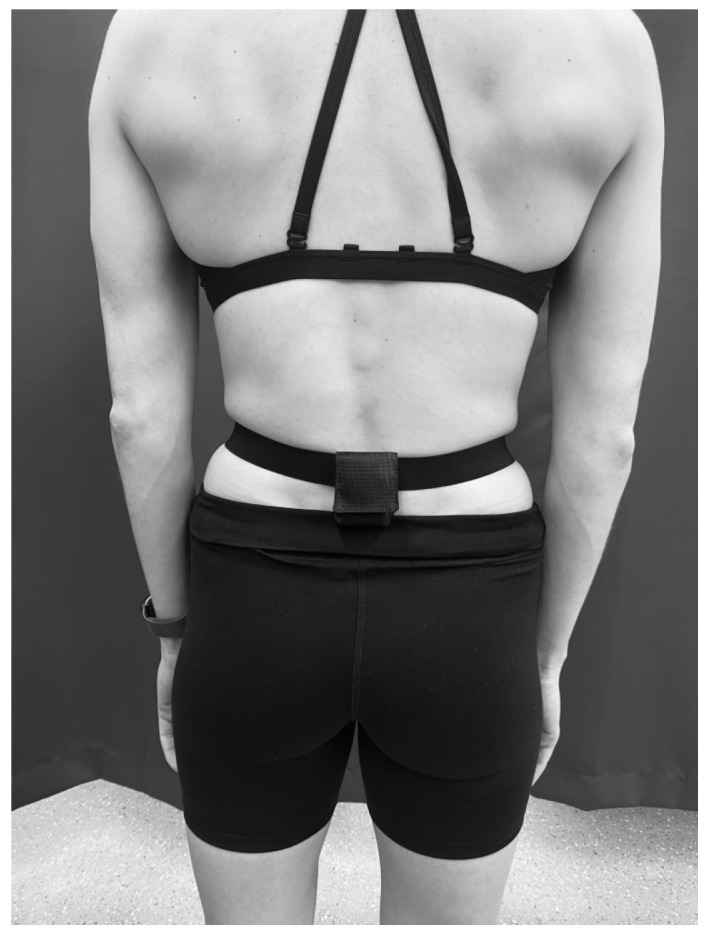
Belt worn around the waist at L4–L5.

The belt-mounting method involved attaching an accelerometer to the inside surface of a latex-free pouch (ActiGraph, Pensacola, FL, USA), using double-sided sticky tape, which was then attached to a latex-free elastic belt (ActiGraph, USA). The belt was worn around the participant’s waist, with a snug fit, around the lumbar spine (L4–L5 level) (see Figure 2).

### 2.4. Anthropometric Measurements

The participant’s height was measured without shoes to the nearest 0.1 centimetre (cm), using a stadiometer (Seca 799). Body mass (BM) was measured in kilograms (kg) (Seca 799) in everyday clothing. A clothing allowance of 0.5 kg was provided. Body mass index was calculated using the following equation: weight (kg)/height squared (m^2^).

### 2.5. Part 1 Procedure

In part 1, the validity of the belt-mounted method was examined in controlled activities conducted in the laboratory and daily activities conducted in a free-living environment, with the procedures described below.

#### 2.5.1. Controlled Activities

**Figure 3:**
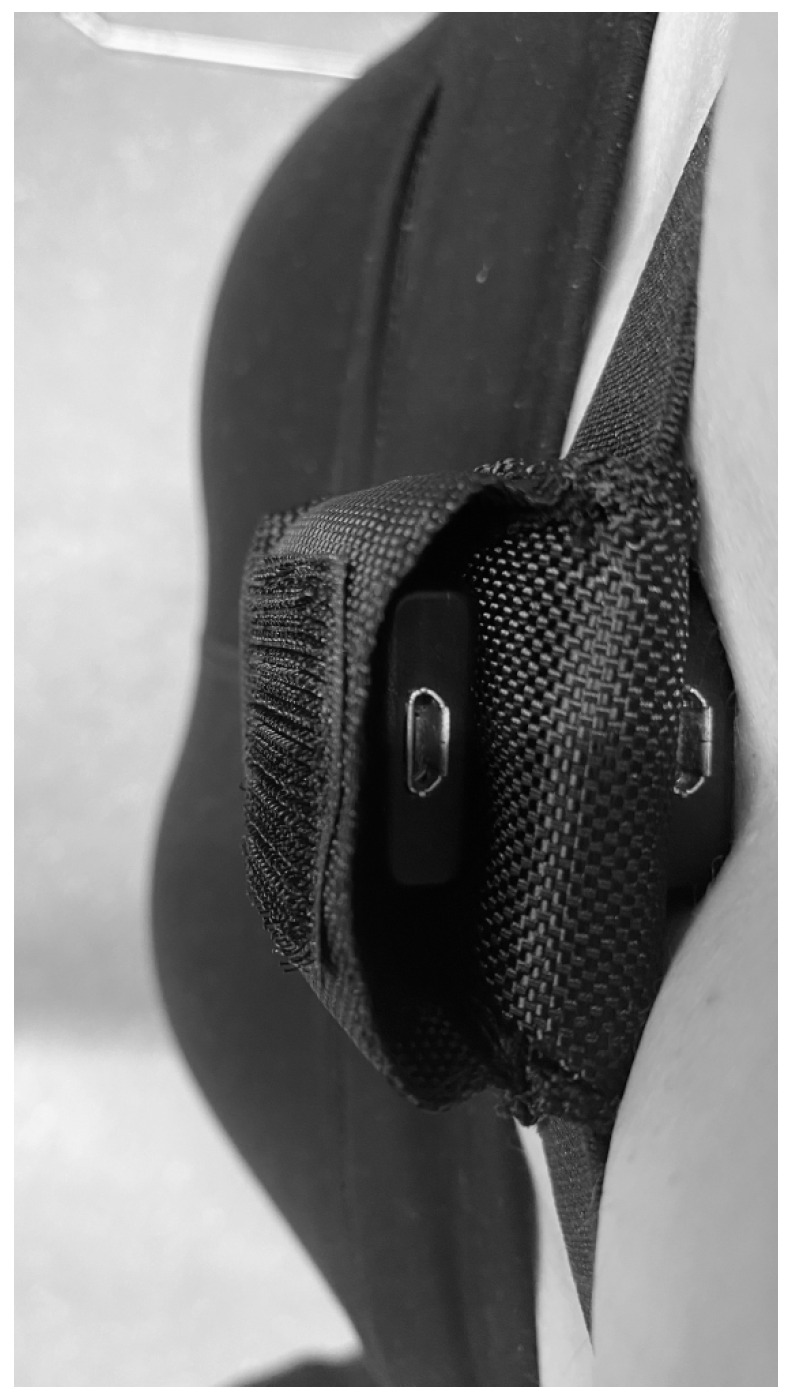
The stacking position of the accelerometers during the controlled activities and daily living conditions in part 1.

During the configuration of the accelerometers in the laboratory, the accelerometers were set to start recording when the accelerometer was disconnected from the USB port. The researcher noted the start time of each of the controlled activities to ensure the data for each individual activity could be distinguished during analysis. The researcher attached the accelerometers to the participants, using the two mounting methods. Participants wore both accelerometers simultaneously during the activities. The belt-mounted accelerometer was worn over the top of the skin-mounted accelerometer so that both accelerometers were at the same position on the lower back (see Figure 3). Participants completed eight controlled activities consisting of slow, normal, and fast walking, slow, normal, and fast running, and descending and ascending four flights of stairs. The walking and running activities were performed on a treadmill. Each of these activities lasted approximately thirty seconds. The speed set on the treadmill for each activity was as follows: slow walking (SW) (3.4 km/h), normal walking (NW) (4 km/h), fast walking (FW) (5 km/h), slow running (SR) (6 km/h), normal running (NR) (6.5 km/h), and fast running (FR) (8 km/h). Descending and ascending the stairs were self-paced by the participants. These speeds were chosen to allow all the participants to comfortably complete the activities without the activities being too strenuous for participants with low fitness levels. After completing each of the activities, the participants were required to wait for a period of one minute before progressing onto the next controlled activity (e.g., normal walking). This was performed to ensure that all the activities could be distinguished in both sets of accelerometer data.

#### 2.5.2. Daily Activities Monitoring

This protocol involved wearing the accelerometers using both mounting methods simultaneously for a whole day in free-living environments for approximately 12 h. The accelerometers were configured to start recording data at around 7 a.m. and to stop recording at around 9 p.m. on the testing day. To ensure the researcher could accurately identify the length of time the accelerometers were worn, the participants were instructed to record the time they put the accelerometers on and the time they removed them at the end of the day. If the accelerometer mounted via the tape method fell off during the day, the data collected would not be used, and the participants were asked to complete another full day of activity monitoring to ensure that the chance of missing data between both methods was limited. The instance of the tape-mounted accelerometer becoming detached during the day occurred in 20% of the participants. As such, the data on these occasions was not used, and these participants were required to complete the full day of activity monitoring again.

Participants were instructed to wear the accelerometers in the same way they wore them during the controlled activities. The researcher asked the participants to practice applying both methods in the laboratory and checked for understanding until the participants were confident that they could correctly apply both methods by themselves. An instruction sheet was also provided to participants to guide them on how to wear both accelerometers properly while they were away from the laboratory.

### 2.6. Part 2 Procedure

This protocol required testing the participants in the laboratory on two visits, one week apart, repeating only the controlled activities. The procedure for each visit is described below. The accelerometers were configured in the same manner as in part 1. On the first visit, participants were required to read a set of instructions on how to apply the belt around their waist at the correct lumbar spine level (L4–L5). As they understood from the instructions, participants attached the belt around their waist without any interference from the researcher. To check for the reliability of the instructions, the position of the belt was checked and the researcher noted if the belt was applied in the correct position. The researcher did not inform the participant if the belt was positioned correctly. The testing protocol of the controlled activities from part 1 was then implemented with the participant. The participant removed the belt after all the controlled activities were completed. The researcher then attached a new accelerometer directly to the participant’s skin at the correct position (L4–L5), using double sided, hypoallergenic sticky tape. The testing protocol from part 1 for the controlled exercises was repeated.

On the second visit, participants repeated the same protocol as they did on their first visit. The researcher did not inform the participants if they had applied the belt method in the correct position until after all the testing protocols and data collection over the 2 days were completed. At the end of the study, participants were provided with a debriefing sheet.

### 2.7. Data Analysis

#### 2.7.1. Physical Activity Measures

The accelerometer data were downloaded using AX3 GUI and then analysed using a customised MATLAB programme (v.7.10.0, R2013a). The customised programme first calculated the resultant acceleration, which was then filtered using a Butterworth bandpass filter (0.1 to 6 Hz) in order to remove the static gravitational acceleration and noise. It was suggested that bone accelerations can only be reliably measured on the skin of the lumbar spine for a frequency of up to around 6 Hz, as the natural frequency of the covering skin and soft tissues was measured at around 10 Hz [31]. Therefore, a cutoff frequency of 6 Hz was employed in this study to filter the acceleration signals. The computed acceleration data were divided into consecutive segments with a length of five seconds (s) each. A Fast Fourier transformation was completed at each segment to ascertain the Fourier series in frequency domain. The loading intensity of the activities, normalised to body weight (BW), was calculated at each segment as follows:

$$LI\;=\;\sum_{fi=0.1}^{6Hz}\frac{\left(Ai\times fi\right)}{g}$$

where LI is loading intensity with the unit of body weight per second (BW/s), Ai is acceleration (m/s^2^) at frequency fi, and g is gravitational acceleration (9.81 m/s^2^).

Loading intensity values calculated from consecutive segments of a controlled activity were averaged to obtain the loading intensity for that activity. There were 8640 segments in total (each 5 s long) in 12-h long free-living activities. Each of these segments was categorised according to its loading intensity into one of the following: very light activity (less than 5 BW/s), light activity (5–10 BW/s), moderate activity (10–15 BW/s), or vigorous activity (over 15 BW/s). The duration of PA spent in a specific category was calculated by multiplying the number of segments within the category with the duration of each segment (5 s). The loading dose of PA was calculated at each of the four intensity categories as follows:

$$LD\;=\;\sum_{k}\;5\times LI$$

where LD is the loading dose at a specific intensity category with the unit of body weight (BW), k is the number of 5 s segments belonging to a specific intensity category, and LI is the loading intensity. More details on the calculation of the loading intensity and loading dose can be found in the previous publication [13].

#### 2.7.2. Statistical Analysis

As data for some variables are not normally distributed according to a one-sample Kolmogorov–Smirnov test, non-parametric statistical analysis methods were used for statistical analysis. For part 1, the Wilcoxon test was employed to examine the effects of the mounting method on variables of controlled activities and daily activities. The inter-rater reliability of the belt and tape method measurements were assessed using the intraclass correlation coefficient (ICC). A two-way mixed model was used, with absolute agreement definition and confidence intervals set at 95%. Bland–Altman plots were calculated by the belt condition subtracted from the tape condition and were used to analyse agreement and evaluate any bias between the two methods.

For part 2, the Wilcoxon test was employed to examine the difference in loading intensity measures for each of the controlled activities between test days (visit 1 and visit 2).

To examine whether putting two accelerometers together in part 1 could induce any interference between the sensors, the loading intensity of the controlled activities measured in part 1 was compared to that measured in part 2, using a repeated measures ANOVA. Mauchley’s test was used to assess sphericity, where a violated Greenhouse Geisser correction was used. Interactions were assessed and pairwise comparisons were treated using the Bonferroni adjustment. A comparison of the individual loading intensity of the controlled activities measured in part 1 were compared to those measured in part 2, using the Mann–Whitney U test for a more in-depth analysis. For these multiple comparisons, the Bonferroni correction was used to adjust the significance level as 0.05/number of comparisons.

All statistical analyses were carried out using the IBM Statistical Package for Social Science (SPSS) version 21 for Windows (SPSS, Chicago, IL, USA). The level of significance was set at *p* < 0.05.

## 3. Results

Ten healthy participants volunteered to participate in each part of this study. The demographics of the participants can be found in Table 1 and Table 2. During the controlled activities, all the skin-mounted accelerometers maintained complete contact with the skin. The researcher checked the skin attachment to ensure it was still securely attached between each of the controlled activities.

### 3.1. Part 1

#### 3.1.1. Controlled Activities

**Figure 4:**
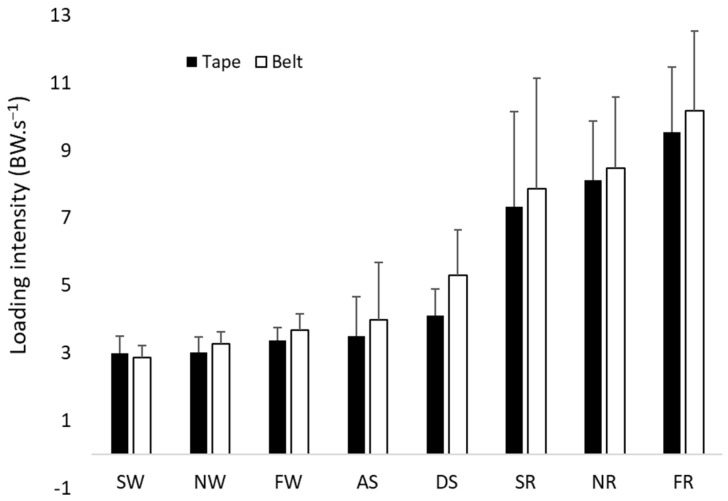
Loading intensities of the belt method (belt) and skin-mounting method (tape), with error bars for SD, SW, NW, FW, SR, NR, FR, ascending stairs (AS), and descending stairs (DS).

There were no significant differences in loading intensity during slow walking (*p* = 0.333), normal walking (*p* = 0.074), fast walking (*p* = 0.047), slow running (*p =* 0.037), normal running (*p* = 0.037), fast running (*p* = 0.093), descending stairs (*p* = 0.011), or ascending stairs (*p* = 0.959) between the belt- and skin-mounting methods, with the adjusted significance level at 0.006 (see Figure 4).

The average ICC measures show good reliability for slow, normal, and fast walking and ascending the stairs. Excellent reliability was shown for slow, normal, and fast running. Descending stairs demonstrated poor reliability when comparing the data captured by the two mounting methods (see Table 3). Bland–Altman plots for the controlled activities demonstrated a consistent negative bias between the tape method and the belt method, suggesting that the belt method consistently records lower loading intensities than the tape method. However, the bias is not significant, as the plots are all within the confidence intervals. The bias also does not increase with the increase in measurements, as shown by the regression analysis. It seems that the bias is within the range of −0.1 to −0.5 BW/s, which is less than 10% (or 5%) of the actual mean loading intensity across most of the controlled activities. However, it seems that ascending and descending stairs had a larger bias between the two methods (maybe more than 10%). Therefore, there is a consistent, but small, bias of using the belt method to measure loading intensity.

#### 3.1.2. Daily Activities Monitoring

There were no significant differences in time spent for very light (*p* = 0.212), light (*p* = 0.149), or moderate physical activity (*p* = 0.074), or dose of very light (*p* = 0.203), light (*p* = 0.059), or moderate physical activity (*p* = 0.080) between the belt- and tape-mounting methods during a whole day measurement, with the adjusted significance level being at 0.008 (see Table 4 and Table 5). None of the participants recorded any vigorous activity.

The Bland–Altman plots showed a mean bias of −9.0 (±28.6) for very light activity with limits of agreement at −65.0 and 47.0, and a mean bias of 13.0 (±28.3) for light activity, with limits of agreement at −42.4 and 68.4, between the two measurements. Moderate activity was not analysed due to the majority of participants not recording any moderate activity. There appears to be a small bias for the duration of very light activity (9 s) and light activity (13 s). However, the bias appears to become larger with the increase in duration, which is the consistent error of loading intensity that accumulates with time (see Figures S1–S8 in the Supplementary Materials).

### 3.2. Part 2

**Figure 5:**
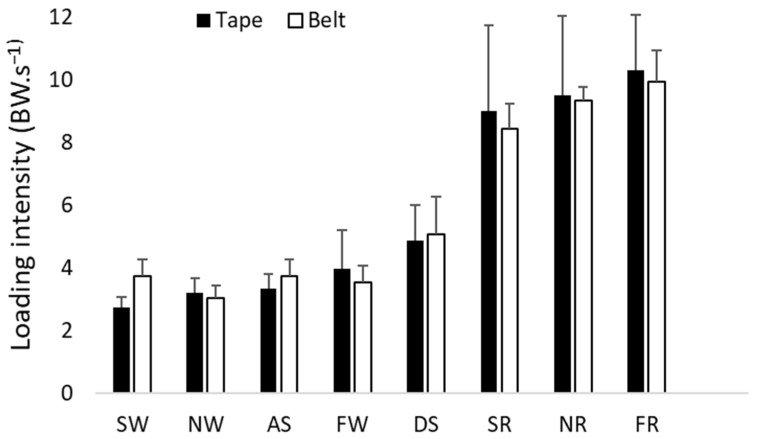
Visit 1: Loading intensities of the belt method (belt) and tape method (tape), with error bars for SD, during SW, NW, FW, SR, NR, FR, AS, and DS.

**Figure 6:**
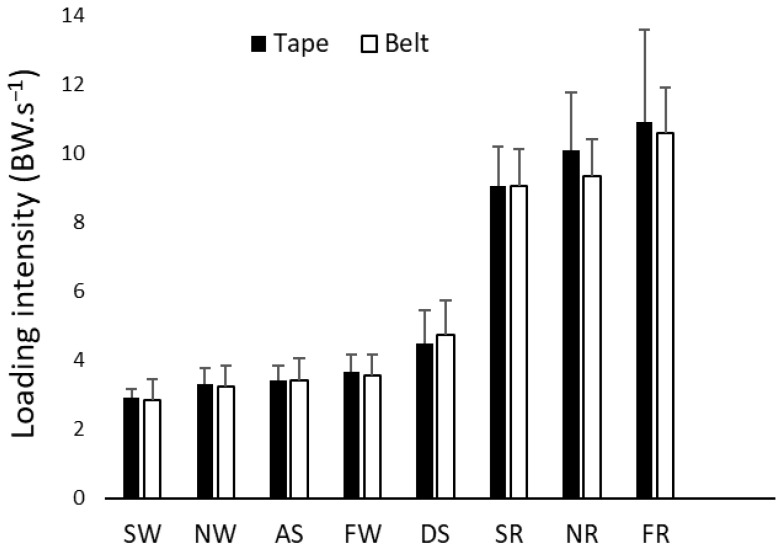
Visit 2: Loading intensities of the belt method (belt) and tape method (tape), with error bars for SD, during SW, NW, FW, SR, NR, FR, AS, and DS.

The results showed no significant differences in loading intensity between the testing days for slow walking (*p* = 0.139), normal walking (*p* = 0.314), fast walking (*p* = 0.721), slow running (*p* = 0.139), normal running (*p* = 0.203), fast running (*p* = 0.374), descending stairs (*p* = 0.484), or ascending stairs (*p* = 0.959) using the skin-mounting method, with the adjusted significance level at 0.006. There were also no significant differences in loading intensity between the test days for slow walking (*p* = 0.878), normal walking (*p* = 0.093), fast walking (*p* = 0.959), slow running (*p =* 0.059), normal running (*p* = 0.878), fast running (*p* = 0.203), descending stairs (*p* = 0.386), or ascending stairs (*p* = 0.114) using the belt-mounting method, with the adjusted significance level at 0.006. Figure 5 and Figure 6 demonstrate the loading intensities of the belt-mounting and skin-mounting methods across the controlled exercises, during visit one and visit two, respectively.

ICC were used to compare the measurements collected by each method on each visit. The measurements collected on visit 1 by the belt were compared against the measurements collected by the belt on visit 2, and vice versa for the tape method. Results indicate that both the belt and tape method had low ICC scores on similar activities (see Table 6 and Table 7). These results demonstrate that for the majority of activities, both the tape and belt method have good reliability and are comparable to each other.

### 3.3. Comparison Between Part 1 and Part 2

The repeated-measures ANOVA demonstrated no significant main effects for mounting the two accelerometers simultaneously (in part 1) (F([1,9]–[0.984], (*p* = 0.347) and mounting the accelerometers individually (in part 2) (F([1,9] = [1.643], (*p* = 0.232). There was a significant interaction between mounting the accelerometers simultaneously and mounting the accelerometers individually (F([1,9] = [5.734], (*p* = 0.040), and post hoc analysis indicated that the mean for the tape-mounted accelerometer was lower during the protocol for the controlled activities in part 1. However, there were no significant interactions between mounting the accelerometer simultaneously and intensity (F([2.023,9] = [1.038], (*p* = 0.375) or mounting the accelerometers simultaneously and mounting the accelerometers individually and loading intensity scores across the controlled activities for both the part 1 and part 2 protocols (F([2.437,63] = [1.018], *p* = 0.391).

There were no significant differences in loading intensity between study 1 and 2 for slow walking (*p* = 0.912), normal walking (*p* = 0.739), fast walking (*p* = 0.684), slow running (*p* = 0.684), normal running (*p* = 0.280), fast running (*p* = 0.280), descending stairs (*p* = 0.684), or ascending stairs (*p* = 0.684) using the belt-mounting method, with the adjusted significance level at 0.006. There were also no significant differences in loading intensity between study 1 and 2 for slow walking (*p* = 0.247), normal walking (*p* = 0.315), fast walking (*p* = 0.143), slow running (*p* = 0.353), normal running (*p* = 0.089), fast running (*p* = 0.123), descending stairs (*p* = 0.247), or ascending stairs (*p* = 0.481) using the skin-mounting method, with the adjusted significance level at 0.006. The average loading intensities collected by either mounting method in part 1 and part 2 are presented in Table S1 in the Supplementary Materials. The table shows that the average scores for loading intensities across the eight activities collected in part 1 and part 2 are similar for the tape method and the belt method.

### 3.4. Reliability of the Instructions

Results showed that 80% of the participants correctly positioned the belt around their waist on their first visit, using the customised set of instructions. Additionally, 90% of participants correctly positioned the belt around their waist on their second visit. The participants who did not position the accelerometer correctly were within 1.5 cm of the correct spinal level, with the accelerometer positioned either lateral of L4–L5 or superior to L4–L5. However, these misalignments did not affect the validity of the data collected, as seen in the results.

## 4. Discussion

This two-part study demonstrates that the belt-mounting method is a reliable and valid method of measuring PA levels in daily activities. With the training and instructions provided, participants could wear the belt-mounted accelerometer at the correct position, which enabled the reliable measurement of PA. There were no significant differences in PA measures between the skin-mounting method and the belt-mounting method in controlled exercise and free-living activities. The ICC average measure results indicate that the belt method demonstrates excellent reliability for slow, normal, and fast running. Moderate reliability was shown for fast running, and good reliability was shown in slow and normal running and ascending stairs. Descending stairs demonstrated poor reliability when comparing the data captured by the two mounting methods. These results suggest that the belt method can be used to measure the overall PA levels, which is comparable to that of the laboratory standard of taping an accelerometer to the spine.

The results of this study indicate that the belt method consistently provides a smaller loading intensity measure than the back method. In practice, this could lead to an overestimation of activity duration or an underestimation of the loading dose (particularly during very light activity), especially as the bias in loading intensity accumulates with time. The current study has several strengths. Firstly, the study assessed the data collected when both mounting methods were simultaneously and singularly applied to the participants, which enabled the direct and fair comparison of the two methods. A set of instructions were used to guide the participant to correctly apply the belt-mounting method by themselves. Our results showed that this is a participant-friendly, convenient, and reliable method to collect data during free-living activities. This method can be used over extended periods of time, with little discomfort or stress to the participant. Our results highlight that the belt-mounting method is comparable to the standard laboratory method of attaching accelerometers by using double-sided adhesives to the skin. To the authors’ knowledge, this is the first study to assess the reliability and validity of using belt-mounted accelerometers to collect PA data. Finally, the current study assessed the PA level using the loading intensity and loading dose. These parameters reflect the amount of mechanical loading experienced by musculoskeletal structures in the lower back [13,32]. They are particularly relevant to low back pain research, as mechanical loading is a critical factor in both the development and persistence of low back pain [10,11,32,33,34,35].

This study’s results on free-living activities are similar to that of Chahal et al. [13], whereby participants spent very little time on moderate and vigorous activity, with over half of the participant sample not recording any vigorous activity data during daily activities. This is also comparable to another study which demonstrates that an acceleration magnitude greater than 3.1 g (moderate levels of activity) is rare when recording activities of daily living [36].

The results of this study on controlled activities are comparable to a previous study [13] which also measured the loading intensity of similar activities. However, it seems that the loading intensity measured in the current study is slightly lower, especially for normal running and fast running. This may be because Kelley et al. [31] asked their participants to complete the eight controlled activities in the natural environment at a self-selected pace, while in this study, the pace for the eight activities was pre-determined for the participants in a laboratory. The walking and running speeds used in this study may have underestimated the actual pace that humans walk and run at in everyday life. Moreover, as the paces were set, there would be no real variability in the data recorded, aside from stride length. This would also provide an explanation for the lower loading intensities reported.

The ICCs for ascending and descending stairs in part 2 indicate that neither mounting method has good reliability. There are a few reasons for this. Firstly, this exercise was self-paced by the participants. Participants may have varied the speed at which they ascended and descended the flights of stairs between visits and when wearing either mounting method. Furthermore, there is an increase in gait variability during the ascent and descent of the stairs, as opposed to walking on a level surface [36,37]. There is also a suggestion that these two types of activity are inherently more unstable than walking on a level surface, whereby postural instability is increased [38]. This could account for some of the individual differences and variability in performing these activities. A previous study has demonstrated that as the decline and incline become steeper, people will naturally adopt a slower gait velocity [37]. Walking at a slow velocity might not be enough for the accelerometer to accurately detect the activity or provide an underestimation of the activity itself, as seen in previous studies [39,40]. Coupled with the self-paced nature of this activity, this could also account for the differences and larger-than-desirable ICC confidence intervals for both the tape method and belt method, respectively.

However, the tape method demonstrated considerably lower ICC scores than the belt method, particularly for stair ascension, whilst the belt method demonstrated good reliability and similar data output between the two visits in part 2. Stair descension produced similar ICC cores for both the tape and belt method for the reliability between the two visits. However, despite the variability in these two activities, both mounting methods had similar ICC scores in the other activities collected on visit 1 and visit 2. This would suggest that both mounting methods are comparable to each other when it comes to measuring physical activity, within the limits of certain activities.

In part 1 of the study, the accelerometers of the two mounting methods were worn simultaneously by the participants. There was a concern that unintentional interference in data collection between the two accelerometers might have occurred due to the accelerometers being pressed snugly against each other towards the participant’s lumbar spine. However, this concern was addressed by part 2, which was designed purposefully to assess any differences in data collection between the skin-mounting method and the belt-mounting method when applied singularly to the participants throughout the controlled activities. There was a significant interaction between data collected for the controlled activities in part 1 and part 2 (*p =* 0.040). This suggests that the stacking of the accelerometers together possibly did cause significant coupling interference or distorted the true difference between the mounting methods: in particular, with the tape-mounted accelerometer, in which a significant difference between the loading intensities was collected compared to the belt-mounted accelerometer. This would suggest that the belt reduced the amount of movement detected by the tape-mounted accelerometer. However, when the data for the controlled activities from part 1 and part 2 were analysed together, results demonstrated there was no significant interaction. This would suggest that there were no significant differences in the loading intensity data collected by the protocols used in part 1 (simultaneous mounting) and part 2 (individually mounting the accelerometer using either method) (*p* = 0.391). This reinforces the finding that the belt-mounting method is a valid method to assess the PA level in both controlled exercises and a free-living environment and it has good day-to-day reliability. Part 2 was also designed to test the reliability of a set of instructions, instructing the participants on how to correctly apply the belt method themselves, without interference from the researcher. As demonstrated in the results, the vast majority (90%) of the participants were able to correctly position the belt. When participants did not correctly apply the belt, displacement of the belt was within 1.5 cm (lateral or superior) of the correct spinal level. However, this did not impact the results of the study, as no significant difference between the mounting methods was shown. This highlights that even if the participants do slightly misposition the belt, the belt method is still a valid mounting method. Therefore, the set of instructions were demonstrated to be reliable, suggesting that they can be used in trials, whereby the participants need to self-apply a belt-mounted accelerometer to monitor activity levels over a prolonged period of time when using the belt-mounting method.

There are several limitations in this study. None of the participants recorded any vigorous activity and only a few managed to record some moderate activity during the whole day assessment. The validity of the belt method during vigorous free-living activity could not be assessed and evaluated as a result of this. However, the results of controlled activities showed that a belt-mounted accelerometer is a valid method to assess vigorous activities such as fast running [13]. The sample size is relatively small, which may have contributed to some of the wide range of limits of agreement seen in the Bland–Altman plots for both the controlled activities and free-living activity monitoring. However, the limits of agreement were very small for the duration of very light activities, which is around 112 s (−65.0 to 47.0) for a whole day, with the average total duration of such activity being around 43,000 s. On the other hand, although the limits of agreement for light activity were at a similar level (−42.4 to 68.4 s), it was relatively higher when compared with the average time of such an activity recorded during a day (only around 100 s). Therefore, further study is needed to examine the validity of the belt-mounted method using participants who would engage in longer durations of moderate and vigorous activities.

The wide limits of agreement for both the duration of and loading dose of light activity could be due to the participants not engaging in a lot of this type of activity in their daily life, as it was found that the average time spent in such an activity across all the participants was approximately 110 s in a whole day, with some participants not even recording any time spent undertaking this type of activity. Therefore, it is expected that there would be wide limits of agreement between the two mounting methods in this activity domain. On the other hand, the total average time spent engaging in very light activity was approximately 43,000 s for each participant. The limits of agreement for this activity were much smaller, being about a 0.2% difference between the two mounting methods. Future research into the differences between these two mounting methods would need to recruit either a larger sample size or include controlled activities that would exceed the threshold for light and moderate activity to collect more data over a longer length of time.

Another limitation is that the free-living activities were only assessed for one day. However, the instructions provided to participants were able to guide them to wear the belt-mounted accelerometers reliably in free-living environments. It is reasonable to suggest that the set of instructions should enable a reliable assessment of PA for a more extended period, but this requires further investigation.

### Clinical Implications and Future Research

The results of these studies would benefit future LBP research where data collection, using accelerometers, is consistently required over a long period of time (e.g., several weeks). It was reported by the participants in part 1 that the accelerometer attached to the lumbar spine with double-sided hypoallergenic sticky tape fell off on two occasions for two different participants during the full day of activity monitoring, due to human sweat and prolonged movement. This would not be ideal and would interfere with the integrity of the data collected in an LBP study and possibly make the participant uncompliant, if the participant was to keep on having to re-apply the accelerometer to their lumbar spine, or if the accelerometer was to be lost after falling off the back of the lumbar spine. This would result in lost/missing data, which would impact the results and outcomes of the trial. The belt method would address these issues, as there is a minimal chance of the accelerometer being lost, and the belt is quick and easy to apply. Future studies should look at the differences in data collection using alternative mounting methods during light, moderate, and vigorous activity and over several days to fill this gap in the literature. The belt-mounting method can also be used in future trials wishing to establish the dose–response effect that quantities of activity have on the outcomes of back pain, such as pain levels or perceived disability.

## 5. Conclusions

This two-part study has demonstrated that the belt-mounting method of wearing an accelerometer around the lumbar spine is a reliable and valid method to assess daily PA. This method is comparable to the laboratory standard of mounting the accelerometer to the lumbar spine via double-sided hypoallergenic adhesives. This study provides researchers with an alternative method of mounting the accelerometer for the monitoring of PA data collection, especially for studies that are designed to monitor activity daily over a long period of time.

## Figures and Tables

**Table 1:** Demographics of the participants included in part 1.

Participant Demographics—Part 1	
Characteristics	Mean ± Standard Deviation (SD)
Age	31.8 ± 10.75
Height (cm)	171.8 ± 11.91
Weight (kg)	76.1 ± 16.4
BMI (kg/m^2^)	25.3 ± 4.8

**Table 2:** Demographics of the participants included in part 2.

Participant Demographics—Part 2	
Characteristics	Mean ± SD
Age	37.7 ± 15.98
Height (cm)	172.8 ± 11.9
Weight (kg)	81.2 ± 10.1
BMI (kg/m^2^)	27.6 ± 3.7

**Table 3:** ICC results between the tape and belt method for the controlled activities.

Controlled Activities	ICC Scores	Confidence Intervals (CI)	Significance Level (*p*-Value)
SW	0.718	[−0.066 to 0.929]	0.036
NW	0.778	[0.055 to 0.946]	0.005
FW	0.676	[−0.127 to 0.916]	0.022
SR	0.976	[0.880 to 0.994]	<0.001
NR	0.974	[0.867 to 0.994]	<0.001
FR	0.906	[0.625 to 0.977]	<0.001
AS	0.765	[0.135 to 0.940]	0.019
DS	0.218	[−0.632 to 0.748]	0.295

**Table 4:** Means and standard deviation for loading dose of free-living activities.

Category of Activity	Mounting Method	Loading Dose (BW) Mean ± SD
Very light	Belt Tape	13,921.3 (±5989.7) 14,074.6 (±6128.4)
Light	Belt Tape	765.4 (±1021.4) 649.3 (±772.9)
Moderate	Belt Tape	125.7 (±339.7) 171.2 (±369.7)

**Table 5:** Means and standard deviation for duration of free-living activities.

Category of Activity	Mounting Method	Duration of PA (In Seconds) Mean ± SD
Very light	Belt Tape	43,022.5 (±151.9) 43,031.5 (±129.5)
Light	Belt Tape	106.5 (±128.6) 93.5 (±103.9)
Moderate	Belt Tape	11 (±29.7) 15 (±32.5)

**Table 6:** ICC results comparing the measurements collected on visit 1 and visit 2, using the belt method.

Controlled Activities	ICC Scores	Confidence Intervals (CI)
SW	0.453	[−1.671 to 0.87]
NW	0.854	[0.435 to 0.963]
FW	0.894	[0.557 to 0.974]
SR	0.675	[−0.102 to 0.915]
NR	0.762	[−0.041 to 0.942]
FR	0.616	[−0.269 to 0.899]
AS	0.644	[−0.176 to 0.906]
DS	0.477	[−1.162 to 0.871]

**Table 7:** ICC results comparing the measurements collected on visit 1 and visit 2, using the tape method.

Controlled Activities	ICC Scores	Confidence Intervals (CI)
SW	0.588	[−0.344 to 0.891]
NW	0.877	[0.539 to 0.969]
FW	0.856	[0.337 to 0.968]
SR	0.497	[−1.024 to 0.875]
NR	0.871	[0.52 to 0.967]
FR	0.885	[0.567 to 0.971]
AS	0.043	[−4.39 to 0.778]
DS	0.365	[−1.556 to 0.842]

## Data Availability

The data that support the findings of this study are available from the corresponding author, [SW], upon reasonable request.

## Supplementary Materials

The following supporting information can be downloaded at: https://www.mdpi.com/article/10.3390/s26020429/s1, Figure S1: Bland Altman plot comparing slow walking measurement obtained via the tape method versus the belt method, with limits of agreement at –0.67 to 0.93; Figure S2: Bland Altman plot comparing normal walking measurement obtained via the tape method versus the belt method, with limits of agreement at –0.82 to 0.33; Figure S3: Bland Altman plot comparing fast walking measurement obtained via the tape method versus the belt method with limits of agreement at –1.03 to 0.43; Figure S4: Bland Altman plot comparing slow running measurement obtained via the tape method versus the belt method with limits of agreement at –2.12 to 1.04; Figure S5: Bland Altman plot comparing normal running measurement obtained via the tape method versus the belt method with limits of agreement at –1.41 to 0.69; Figure S6: Bland Altman plot comparing fast running measurement obtained via the tape method versus the belt method with limits of agreement at –2.91 to 1.63; Figure S7: Bland Altman plot comparing ascending stairs measurement obtained via the tape method versus the belt method with limits of agreement at –2.95 to 1.99; Figure S8: Bland Altman plot comparing descending stairs measurement obtained via the tape method versus the belt method with limits of agreement at –3.94 to 1.58; Figure S9: Bland Altman plot comparing the measurement of amount of time (in seconds) the participants spent engaging in very light activity obtained via the tape method versus the belt method, with limits of agreement at –65.09 to 47.09; Figure S10: Bland Altman plot comparing the measurement of amount of time (in seconds) the participants spent engaging in light activity obtained via the tape method versus the belt method, with limits of agreement at –42.47 to 68.47; Figure S11: Bland Altman plot comparing the loading dose recorded by the participants for very light activity obtained via the tape method versus the belt method, with limits of agreement at –868.90 to 570.70; Figure S12: Bland Altman plot comparing the loading dose recorded by the participants for light activity obtained via the tape method versus the belt method, with limits of agreement at –382.24 to 604.16; Table S1: Comparisons of the average loading intensity collected by the tape mounted and belt mounted accelerometer in parts 1 and 2 respectively.

## Abbreviations

The following abbreviations are used in this manuscript:

PA	Physical activity
LBP	Low back pain
L4	Lumbar spine level 4
L5	Lumbar spine level 5
LSBU	London South Bank University
AX3	3-axis/triaxial
UK	United Kingdom
USA	United States of America
cm	Centimetres
BM	Body mass
Kg	Kilogram
m^2^	Metres squared
Km/h	Kilometres per hour
hz	Hertz
BW/s	Loading dose per second
m/s^2^	Metre per second squared
fi	Frequency
g	Gravitational force
LD	Loading dose
LI	Loading intensity
ANOVA	Analysis of Variance
ICC	Intraclass Correlation Coefficient
SPSS	Statistical Package for Social Science
SD	Standard deviation
SW	Slow walking
NW	Normal walking
FW	Fast walking
SR	Slow running
NR	Normal running
FR	Fast running
AS	Ascending stairs
DS	Descending stairs
CI	Confidence intervals

## Appendix A

Set of instructions used in part 2:

Thank you for volunteering to participate in this study. Your time and effort are greatly appreciated by the primary researcher and research team.

Below are a set of instructions on how the belt should be worn. There are pictures for further guidance. You do not need to worry about attaching the accelerometer inside the pouch. The primary researcher has done this for you, so all you need to do is fasten the belt around your waist. If you have any questions, please do not hesitate to contact the primary researcher using the contact information at the bottom of this sheet.

1. Feel your lower back for the dimples of your pelvis or feel at around your sides for your hip bones. Once you have located your hip bones, follow the curve of your hip around to your lower back until you locate the bony prominence (feels like flat bone) next to your lower spine (just above your buttocks). Please look at the figure below for guidance.
2. Once you have found the bony prominence, join your hands together on top of the spine. Please place the belt around your waist, with the pouch coming to rest on your lower back. Please see figure below for guidance on how this should look.
3. Ensure the belt is fastened for a snug fit and is worn on the lower part of your hip.
