# Supplementary material for: Validity and Reliability of Using a Belt-Worn Accelerometer on the Lower Back to Monitor Physical Activity
Source: Sensors (Basel). 2026 Jan 9;26(2):429. doi: 10.3390/s26020429 (PMC12845556; doi:10.3390/s26020429)
Supplement: Supplementary file 1 [file sensors-26-00429-s001.zip › sensors-3970019-supplementary.pdf]

Supplementary Materials

Figures:

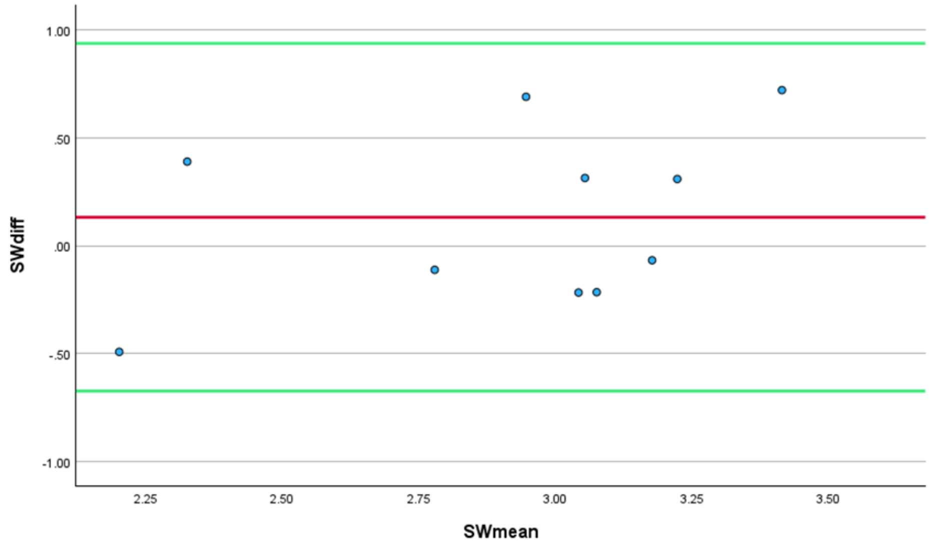

Figure S1: Bland Altman plot comparing slow walking measurement obtained via the tape method versus the belt method, with limits of agreement at –0.67 to 0.93.

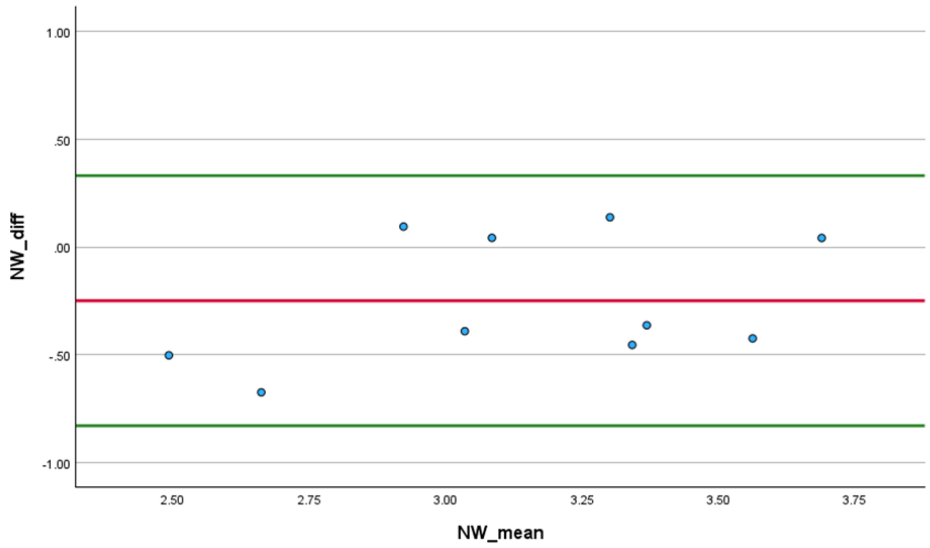

Figure S2: Bland Altman plot comparing normal walking measurement obtained via the tape method versus the belt method, with limits of agreement at –0.82 to 0.33.

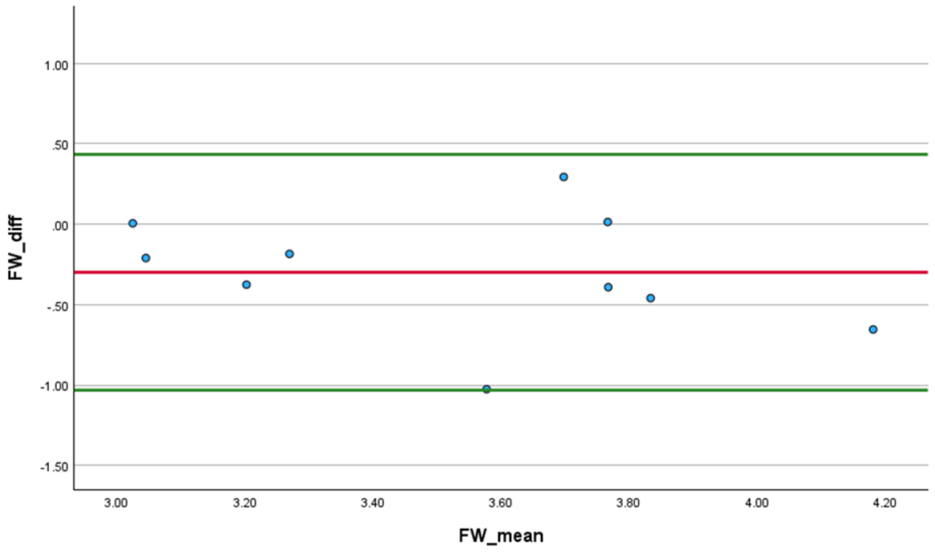

Figure S3: Bland Altman plot comparing fast walking measurement obtained via the tape method versus the belt method with limits of agreement at –1.03 to 0.43.

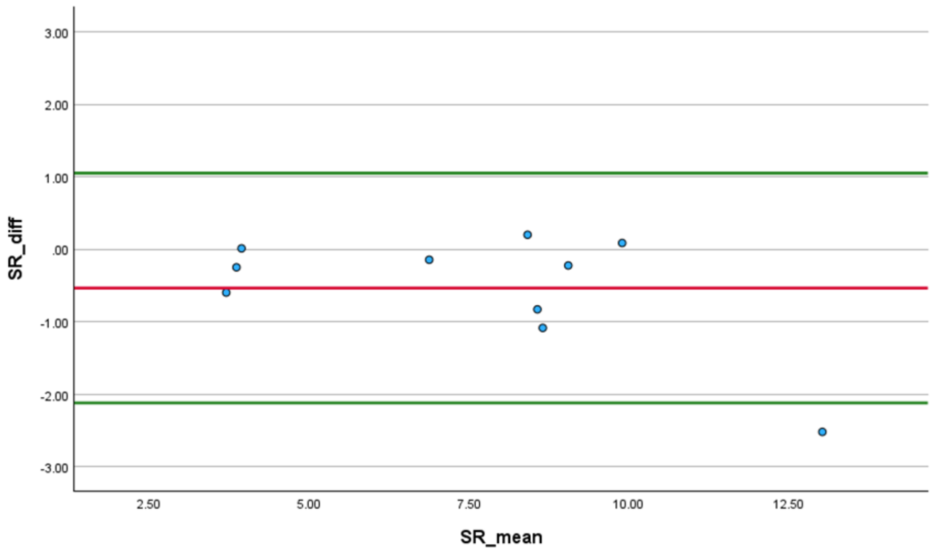

Figure S4: Bland Altman plot comparing slow running measurement obtained via the tape method versus the belt method with limits of agreement at –2.12 to 1.04.

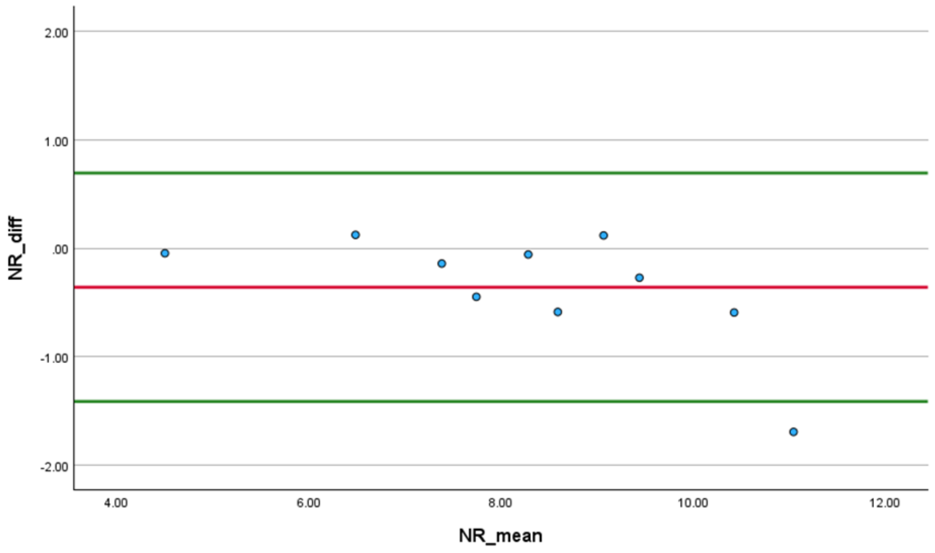

Figure S5: Bland Altman plot comparing normal running measurement obtained via the tape method versus the belt method with limits of agreement at –1.41 to 0.69.

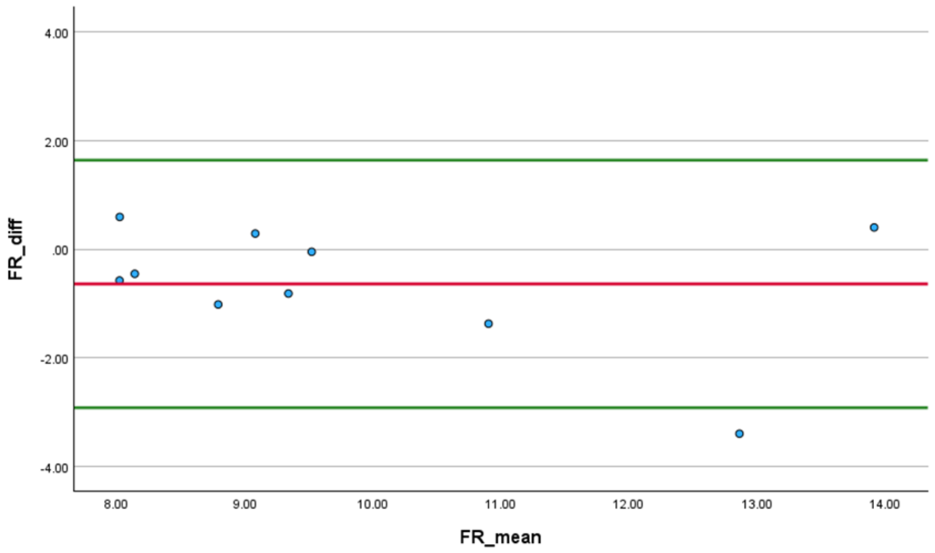

Figure S6: Bland Altman plot comparing fast running measurement obtained via the tape method versus the belt method with limits of agreement at –2.91 to 1.63.

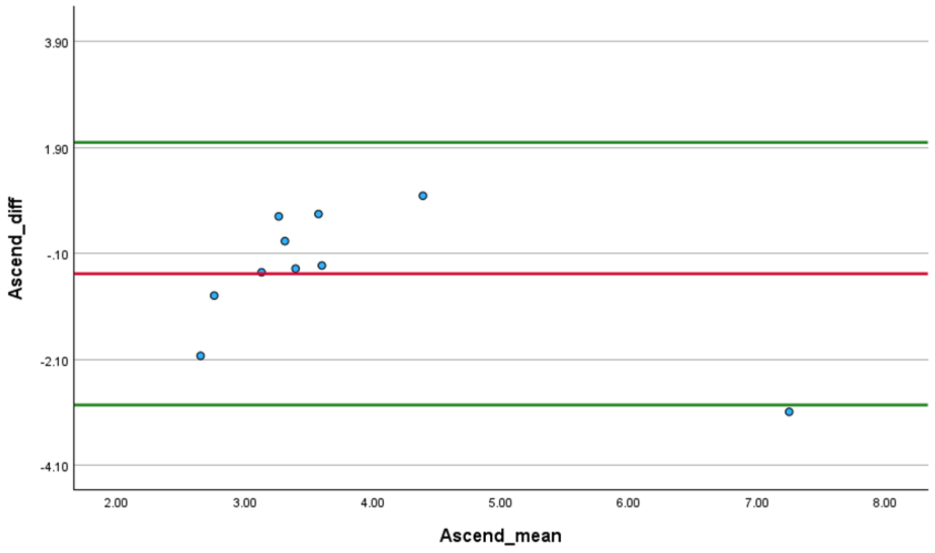

Figure S7: Bland Altman plot comparing ascending stairs measurement obtained via the tape method versus the belt method with limits of agreement at –2.95 to 1.99.

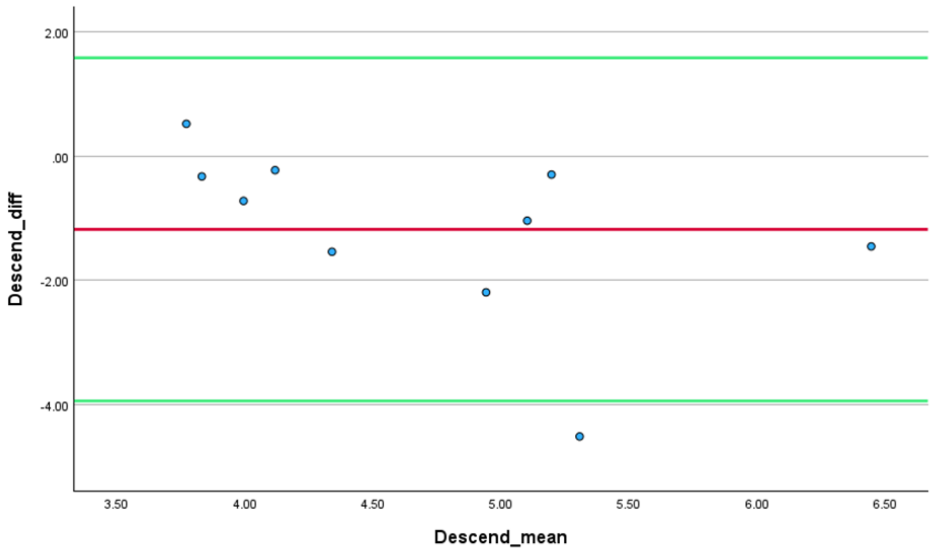

Figure S8: Bland Altman plot comparing descending stairs measurement obtained via the tape method versus the belt method with limits of agreement at –3.94 to 1.58.

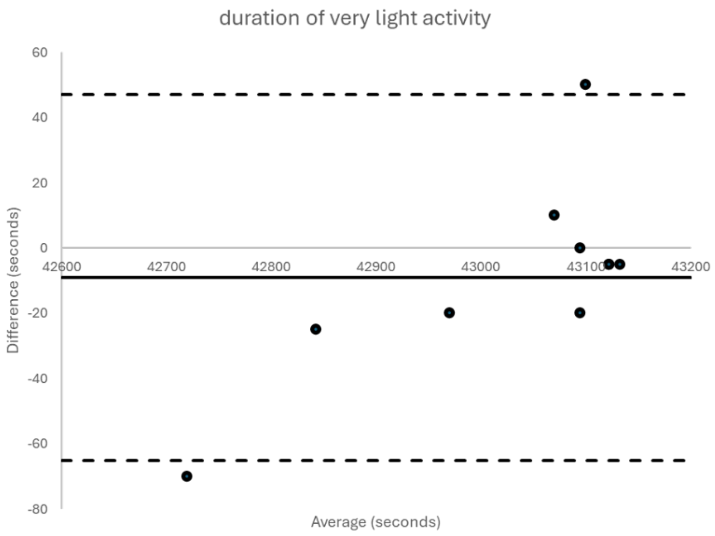

Figure S9: Bland Altman plot comparing the measurement of amount of time (in seconds) the participants spent engaging in very light activity obtained via the tape method versus the belt method, with limits of agreement at –65.09 to 47.09.

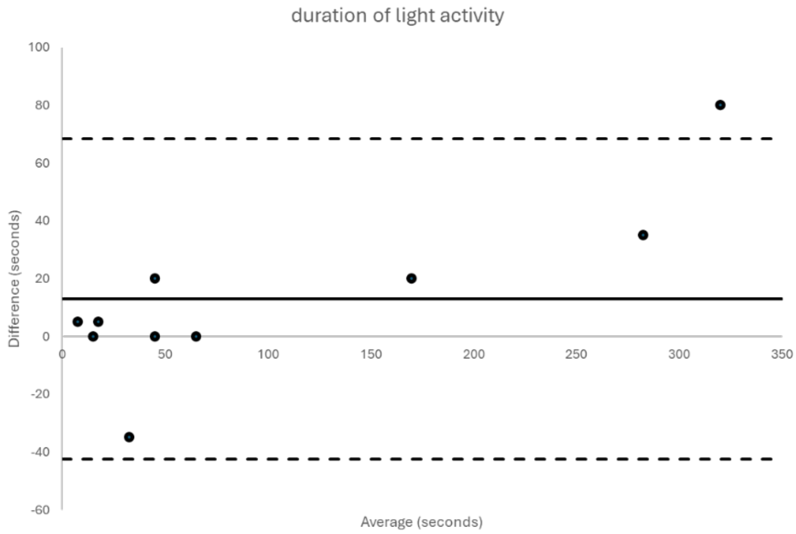

Figure S10: Bland Altman plot comparing the measurement of amount of time (in seconds) the participants spent engaging in light activity obtained via the tape method versus the belt method, with limits of agreement at –42.47 to 68.47.

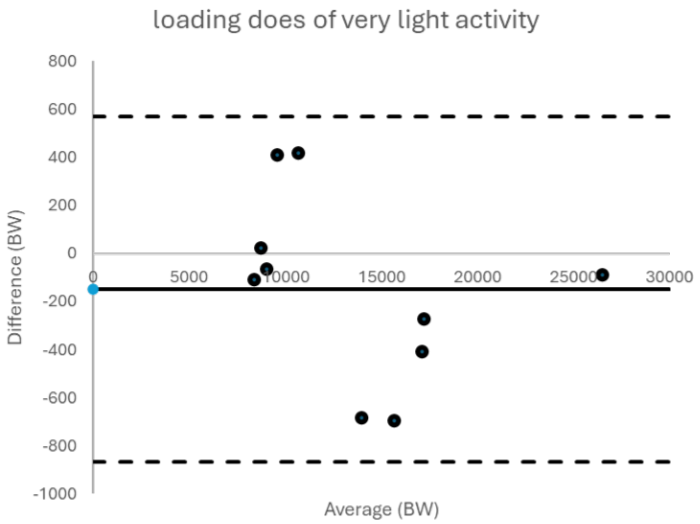

Figure S11: Bland Altman plot comparing the loading dose recorded by the participants for very light activity obtained via the tape method versus the belt method, with limits of agreement at –868.90 to 570.70.

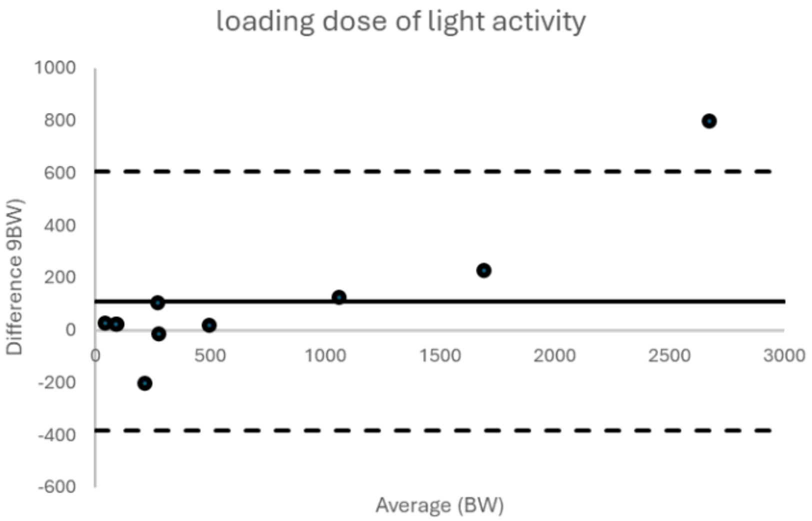

Figure S12: Bland Altman plot comparing the loading dose recorded by the participants for light activity obtained via the tape method versus the belt method, with limits of agreement at –382.24 to 604.16.

Table

Table S1: Comparisons of the average loading intensity collected by the tape mounted and belt mounted accelerometer in parts 1 and 2 respectively.

**Average Loading Intensity**				
Part 1			***Part 2***	
**Controlled Activity**	**Attachment Method**	**Mean ± SD**	**Visit 1 Mean ± SD**	**Visit 2 Mean ± SD**
SW	Belt	2.85 (±.36)	2.87 (±.11)	2.8 (±.19)
	Tape	2.99 (±.51)	2.73 (±.11)	2.9 (±0.84)
NW	Belt	3.3 (±.35)	3.03 (±.14)	3.2 (±.197)
	Tape	3.0 (±.46)	3.2 (±.15)	3.3 (±.146)
Fast walking	Belt	3.69 (±.47)	3.55 (±.18)	3.5 (±.195)
	Tape	3.4 (±.38)	3.98 (±.39)	3.6 (±.166)
Slow running	Belt	7.9 (±3.3)	8.4 (±.267)	9.0 (±.346)
	Tape	7.3 (±2.8)	9.0 (±.864)	9.1 (±.361)
Normal running	Belt	8.5 (±2.1)	9.3 (±.15)	9.3 (±.34)
	Tape	8.1 (±1.8)	9.5 (±.80)	10.1 (±.42)
Fast running	Belt	10.2 (±2.3)	9.94 ± (.328)	10.57 (±.416)
	Tape	9.5 (±1.9)	10.28 (±.56)	10.9 (±.84)
Descending stairs	Belt	5.3 (±1.3)	5.07 (±.397)	4.74 (±.32)
	Tape	4.2 (±1.0)	4.86 (±.360)	4.5 (±.3)
Ascending stairs	Belt	3.7 (±1.2)	3.8 (±.178)	3.4 (±.2)
	Tape	3.6 (±1.3)	3.3 (±.151)	3.4 (±.13)
